# Mycophenolate mofetil versus cyclophosphamide plus in patients with connective tissue disease-associated interstitial lung disease: Efficacy and safety analysis

**DOI:** 10.1515/med-2023-0838

**Published:** 2023-11-16

**Authors:** Pengfei Wang, Li Zhang, Qian Guo, Lifen Zhao, Yanyan Hao

**Affiliations:** Department of Respiratory and Critical Care Medicine, Shanxi Bethune Hospital, Shanxi Academy of Medical Sciences, Tongji Shanxi Hospital, Third Hospital of Shanxi Medical University, Taiyuan, Shanxi, 030032, China; Department of Respiratory, Shanxi Provincial Hospital of Traditional Chinese Medicine, Taiyuan, 030012, China; Department of Respiratory and Critical Care Medicine, Shanxi Bethune Hospital, Shanxi Academy of Medical Sciences, Tongji Shanxi Hospital, Third Hospital of Shanxi Medical University, No.99 of Longcheng Street, Xiaodian District,, Taiyuan, Shanxi, 030032, China

**Keywords:** connective tissue disease, cyclophosphamide, forced vital capacity, Interstitial lung disease, mycophenolate mofetil, prednisolone

## Abstract

The decision for definitive therapy for the treatment of patients with connective tissue disease-associated interstitial lung disease (CTD-ILD) is difficult. Patients with CTD-ILD received 0.5 g twice a day of mycophenolate mofetil for 2 years (MMF cohort, *n* = 105) or cyclophosphamide 50 mg once every other day, and the cumulative dose of cyclophosphamide should not exceed 10 g (CYC cohort, *n* = 140). After complete of treatment (EL), % forced vital capacity (FVC) and % diffusing capacity of the lungs for carbon monoxide were increased in both the MMF and CYC cohorts as compared to before treatment (*p* < 0.001 for all). There were higher changes in % FVC values and a greater number of patients with significant change in % FVC (>10% change) in the CYC cohort than in the MMF cohort (*p* < 0.0001 for both) at EL. Patients in the CYC cohort had higher rates of leukopenia, thrombocytopenia, serious adverse effects related to treatment(s), and death than those in the MMF cohort (*p* < 0.05 for all). Cyclophosphamide plus prednisolone superiorly improved % FVC compared to mycophenolate mofetil plus prednisolone. Mycophenolate mofetil and cyclophosphamide improved pulmonary function. Mycophenolate mofetil is less toxic and increased patient survival.

## 1 Introduction

Interstitial lung disease (ILD) is a type of diffuse parenchymal lung disorders associated with morbidity and mortality [1]. Connective tissue disease (CTD) can affect the components of intestinal lung diseases that are progressive and fatal diseases [2]. Cyclophosphamide is an immunosuppressant that is reported effective in autoimmune and inflammatory illnesses including remission [3]. Cyclophosphamide is an alkylating agent that causes cross-linkage of DNA and the other a variety of macromolecules, bone marrow suppression, and impairs humoral and cellular immune responses [2]. These actions lead to several toxicities associated with the use of cyclophosphamide and are associated with the total cumulative dose [4]. Therefore, the long-term cyclophosphamide treatment is limited to less than 12 months [5]. Mycophenolate mofetil is currently used in organ transplants [6] and in the treatment of autoimmune diseases [7] because of its immunosuppressive properties and favorable safety profile [5]. It is equally effective and safe as cyclophosphamide after 2 years of usage in patients with systemic sclerosis associated ILD [5,8]. In addition, mycophenolate mofetil is well tolerated, has a low discontinuation rate, and has improved pulmonary functions over 2.5 years in patients with connective tissue disease-associated interstitial lung disease (CTD-ILD) [9]. Both mycophenolate mofetil and cyclophosphamide are effective and safe for systemic sclerosis-associated ILD [10]; however, mycophenolate mofetil is not approved in patients with CTD-ILD in China.

The decision for definitive therapy for the treatment of patients with CTD-ILD is difficult [11] because clinicians need to identify patients who will develop progressive disease and balance the need for high levels of therapy in the critically ill patient cohort with the potential adverse effects, because for such conditions a relatively limited literature is available [12].

The objective of this retrospective study was to evaluate the efficacy and safety of 2 years of mycophenolate mofetil plus prednisolone treatment against cyclophosphamide (50 mg once every other day, and the cumulative dose of cyclophosphamide should not exceed 10 g) plus prednisolone treatment in patients with CTD-ILD.

## 2 Materials and methods

### 2.1 Study design

This is a multi-center retrospective analysis. An anonymous data sharing was done.

### 2.2 Ethics approval and consent to participate

The designed protocol of the established study was approved by the Shanxi Bethune Hospital review board (approval number: YXLL-2023-144). The study followed the law of China and the v2008 Declarations of Helsinki. The requirement for informed consent was waived by the Shanxi Bethune Hospital Review Board.

### 2.3 Study subjects and data collection

#### 2.3.1 Inclusion criteria

Patients (≥18 years of age) with CTD-ILD (diagnosed according to the British Thoracic Society guidelines [13]) require to start immunosuppressive treatment(s) and treated with oral cyclophosphamide or oral mycophenolate mofetil were included in the study.

#### 2.3.2 Exclusion criteria

Patients with CTD-ILD who discontinued cyclophosphamide or mycophenolate mofetil and those with incomplete data in institutional records were excluded from the analysis.

### 2.4 Treatment(s)

A total of 105 patients received 0.5 g of oral twice a day of mycophenolate mofetil for 2 years (MMF cohort) and 140 patients received oral cyclophosphamide 50 mg once every other day, and the cumulative dose of cyclophosphamide should not exceed 10 g (CYC cohort) [14]. In addition to mycophenolate mofetil or cyclophosphamide, all patients received oral prednisolone. We first give a moderate dose of prednisone and then reduce the dose after the condition is stable. For example, the dosage of prednisolone was 40–80 mg/day first, which was reduced to 30 mg/day at about 1 month, then reduced by 5 mg every month, and reduced to 20 mg/day after 2 months. After that it was reduced by 2.5 mg every 2 months, and to 15 mg/day after 4 months, after which the reduction may be slower later. It was the same in MMF and CYC treatment.

### 2.5 Outcome measures

#### 2.5.1 Diagnosis of CTD-ILD

CTD-ILD was diagnosed according to the British Thoracic Society guidelines [13]. The presence of specific autoantibodies or histopathology and high-resolution chest computed tomography (OP and a ground-glass opacification (GGO) were pattern of high-resolution chest computed tomography) was performed for the diagnosis of CTD-ILD [15]. >20% lung involvement on high-resolution chest computed tomography did.

The average time of lung function after treatment was one month. The collection of lung functions was after complete of treatment (1 year ± 1 month for CYC cohort and 2 year ± 1 months for MMF cohort; after complete of treatment [EL]). The demographic and clinical conditions of patients, pulmonary physiology before treatment(s) (BL) and EL, and adverse effects were collected from the hospital records of patients and analyzed.

#### 2.5.2 Pulmonary function tests

##### 2.5.2.1 Forced vital capacity (FVC)

FVC is the total amount of air exhaled during forced expiration. A spirometer was used to measure the FVC.

##### 2.5.2.2 Diffusing capacity of the lungs for carbon monoxide (DLCO)

It is a measurement of the lung’s ability to transfer gas from inspired air to the bloodstream. A spirometer was used to measure the DLCO.

Pulmonary function tests were performed by standard spirometry according to the American Thoracic Society (ATS)/European Respiratory Society (ERS) recommendations. The DLCO was measured using the single-breath method (Jaeger, Viaen, Germany). Values were expressed as percentage of the predicted values. A significant change in pulmonary function tests was defined as >10% change in % FVC or >15% change in % DLCO, in conformity with the ATS/ERS guideline.

### 2.6 Adverse effects

Unwanted adverse effects during the treatment and follow-up periods were collected from medical records of patients file and analyzed. Hospitalization or life-threatening effects were considered serious adverse effects related to treatment(s). Hospitalization or life-threatening effects due to persistent disease was considered serious adverse effects related to underlying disease(s).

### 2.7 Statistical analysis

InStat 3.01 (GraphPad Software, San Diego, CA, USA) was used for statistical analysis. Categorical variables are presented as frequencies, with percentages in parentheses. Continuous variables are presented as mean ± standard deviation (SD) if they are normally distributed or as median with Q3–Q1 in parentheses if they are not normally distributed. Fisher’s exact test (in the four-grid table, when the total number of cases was <40 and the theoretical frequency of all grids was less than 5) or chi-square test (*χ* ^2^-test; in the four-grid table, when the total number of cases was ≥40 and the theoretical frequency of all grids was greater than or equal to 5) was used for the statistical analysis of categorical variables. The normality of the parameters was evaluated using the Kolmogorov and Smirnov methods. The paired *t*-test, unpaired *t*-test, or one-way analysis of variance (ANOVA) was used for statistical analysis of continuous variables that were normally distributed with equal SDs. For normally distributed continuous variables with unequal SDs, a paired *t*-test or unpaired *t*-test with Welch’s correction was used for statistical analysis. The equality of the SDs was evaluated using an *F*-test. For non-normally distributed continuous variables, the Wilcoxon matched-pairs signed-ranks test, Mann-Whitney test, or Kruskal–Wallis’ test (nonparametric ANOVA) was used for statistical analysis. The Tukey–Kramer or Dunn’s multiple comparison test was used for *post hoc* analysis. All results were considered significant if the *p*-value was less than 0.05.

## 3 Results

### 3.1 Study population

**Figure 1:**
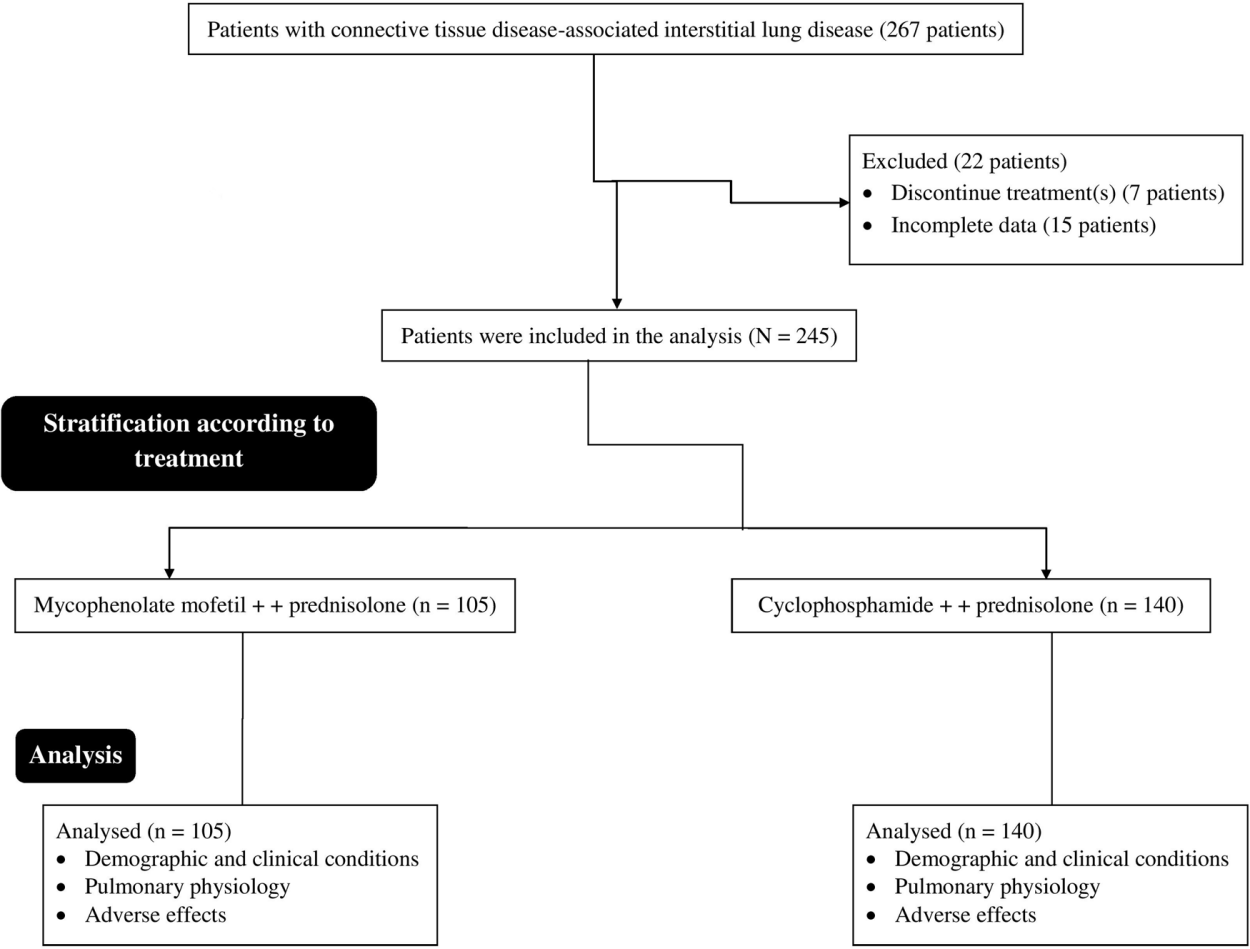
The summary chart of the study.

Between January 15, 2017, and February 12, 2020, 267 Han Chinese patients (≥18 years of age) were diagnosed with CTD-ILD at the Shanxi Bethune Hospital, Shanxi Academy of Medical Sciences, Tongji Shanxi Hospital, Third Hospital of Shanxi Medical University, Taiyuan, China, and the Shanxi Provincial Hospital of Traditional Chinese Medicine, Taiyuan, China. Among them, seven patients discontinued treatment (cyclophosphamide or mycophenolate mofetil) for no reason(s). Complete data for 15 patients were not available at the institutes (≥3 variables). Therefore, data of these patients (22 patients) were excluded from this study. Data on the demographic and clinical conditions of patients at BL, pulmonary physiology at BL and EL, and adverse effects in 245 patients with CTD-ILD were included in the analysis. A summary of this study is shown in Figure 1.

### 3.2 Demographic and clinical conditions

Age, sex, smoking habits of patients, CTD, and pulmonary physiology among cohorts were comparable at BL (*p* > 0.05 for all). A total of 29 (28%) and 40 (29%) patients had FVC ≥70% at BL in the MMF and CYC cohorts, respectively. At BL, 5 (5%) and 10 (7%) patients had 75% or more DLCO in the MMF and CYC cohorts, respectively. None of the patients had ≤40% DLCO at BL. None of the patients had pulmonary hypertension (pulmonary artery systolic pressure >50 mm Hg measured by cardiac color ultrasound, or mean pulmonary artery pressure >20 mm Hg measured by right heart catheterization) at BL. The demographic, clinical, and spirometry parameters of the patients at BL are presented in Table 1.

**Table 1:** Demographic, clinical, and spirometry parameters of patients before treatment(s)

Parameters		Cohorts		Comparisons between cohorts			
		MMF	CYC				
Treatment		Mycophenolate mofetil + prednisolone	Cyclophosphamide + prednisolone				
Patients		105	140	*p*-value	df	Test value	95% CI
Age (years)		56.96 ± 4.78	57.47 ± 5.59	0.4437 (unpaired *t*-test with Welch correction)	238	0.7672	−0.7987 to 1.818
Gender	Male	61(58)	82(59)	0.9404 (*χ* ^2^-test with Yate’s correction)	1	0.0056	0.7378 to 1.325
	Female	44(42)	58(41)				
Smoking habits	Never	90(86)	124(89)	0.4889 (*χ* ^2^-test of Independence)	2	0.4889	N/A
	Previous	15(14)	15(10)				
	Current	0(0)	1(1)				
CTD	Rheumatoid arthritis	26(25)	38(26)	0.7703 (*χ* ^2^-test of Independence)	6	3.301	N/A
	Sjögren disease	25(24)	33(24)				
	Polymyositis/dermatomyositis	20(19)	29(21)				
	Lung-dominant connective tissue disease	13(12)	18(13)				
	Systemic lupus erythematosus	9(9)	14(10)				
	Systemic sclerosis	8(8)	4(3)				
	Mixed connective tissue disease	4(4)	4(3)				
Pulmonary physiology	% FVC	64.85 ± 7.22	64.8 ± 7.95	0.9615 (unpaired *t*-test)	243	0.04827	−1.991 to 1.896
	Numbers of patients with ≥70% FVC	29(28)	40(29)	0.9836 (*χ* ^2^-test with Yate’s correction)	1	0.0004	0.7033 to 1.347
	% DLCO	62.27 ± 8.72	63.22 ± 8.6	0.3934 (unpaired *t*-test)	243	0.8551	−1.245 to 3.154
	Numbers of patients with ≥ 75% DLCO	5(5)	10(7)	0.5925 (Fisher test)	N/A	0.7667	0.3691 to 1.592

Variables are depicted as mean ± standard deviation (SD) or frequencies with percentages in parenthesis.

FVC: Forced vital capacity, DLCO: Diffusing capacity of the lungs for carbon monoxide, df: Degree of freedom, CI: confidence interval (using the approximation of Katz. For categorial variable), N/A: not applicable.

All results are considered significant if the *p*-value was less than 0.05.

Test value (Welch’s approximate t -value for unpaired *t*-test with Welch correction, *χ* ^2^-value for *χ* ^2^-test; *t*-value for unpaired *t*-test; relative risk for Fisher test).

### 3.3 Pulmonary function tests

At EL, the % FVC was increased in both the MMF and CYC cohorts as compared to their BL condition. At EL, % FVC was higher in the CYC cohort than in the MMF cohort. At EL, both the MMF and CYC cohorts had higher numbers of patients with 70% or more FVC than their BL condition. At EL, there were a greater number of patients with 70% or more FVC in the CYC cohort than in the MMF cohort. At EL, % DLCO was increased in both the MMF and CYC cohorts compared to their BL condition. At EL, there is no statistically significant difference for % DLCO between the MMF and CYC cohorts. At EL, both the MMF and CYC cohorts had higher numbers of patients with 75% or more DLCO than their BL condition. At EL, the number of patients with ≥75% DLCO was statistically the same in both cohorts. Numbers of patients with significant change in % FVC were higher in the CYC cohort than that of the MMF cohort. Numbers of patients with significant change in % DLCO were higher in the CYC cohort than that of the MMF cohort but statistically same. The pulmonary physiology of the patients at BL and EL is presented in Table 2.

**Table 2:** Pulmonary physiology of patients before treatment(s) and after treatment(s)

Parameters	Cohorts												Comparisons between cohorts at EL			
	MMF						CYC									
Treatment	Mycophenolate mofetil + prednisolone						Cyclophosphamide + prednisolone									
Level	BL	EL	*p*-value	df	Test value	95% CI	BL	EL	*p*-value	df	Test value	95% CI				
Patients	105	105					140	140					*p*-value	df	Test value	95% CI
% FVC	64.85 ± 7.22	70.96 ± 5.24	<0.001 (ANOVA/Tukey test)	349	28.222	N/A	66(71–57.5)	74(79.5–70)	<0.001 (Kruskal-Wallis’ test/Dunn’s multiple comparisons test)	N/A	115.17	N/A	<0.001 (Kruskal-Wallis’ test/Dunn’s multiple comparisons test)	N/A	95.78	N/A
Numbers of patients with ≥ 70% FVC	29(28)	50(48)	0.0044 (*χ* ^2^-test with Yates correction)	1	3.349	0.5243 to 1.015	40(29)	112(80)	<0.0001 (*χ* ^2^-test with Yates correction)	1	72.547	0.2542 to 0.4463	<0.0001 (*χ* ^2^-test with Yates correction)	1	26.658	0.3531 to 0.6144
Changes in % FVC	8.57(11.57–4.55)		N/A	N/A	N/A	N/A	15.07(20–9.79)		N/A	N/A	N/A	N/A	<0.001 (Kruskal-Wallis’ test/Dunn’s multiple comparisons test)	N/A	239.88	N/A
Numbers of patients with significant change in % FVC	N/A	38(36)	N/A	N/A	N/A	N/A	N/A	105(75)	N/A	N/A	N/A	N/A	<0.0001 (*χ* ^2^-test with Yates correction)	1	35.61	0.2978 to 0.5497
% DLCO	62.27 ± 8.72	71.35 ± 8.37	<0.001 (ANOVA/Tukey test)	349	36.9	N/A	63.22 ± 8.6	72.96 ± 7.47	<0.001 (ANOVA/Tukey test)	384	68.029	N/A	>0.05 (ANOVA/Tukey test)	349	56.846	N/A
Numbers of patients with ≥ 75% DLCO	5(5)	47(45)	<0.0001 (Fisher’s exact test)	N/A	0.1519	0.06546 to 0.3526	10(7)	66(47)	<0.0001 (Fisher’s exact test)	N/A	0.2065	0.1148 to 0.3713	0.81 (*χ* ^2^-test with Yates correction)	1	0.0578	0.7073 to 1.267
Changes in % DLCO	15(17.32–11.43)		N/A	N/A	N/A	N/A	15.94(18.58–12.33)		N/A	N/A	N/A	N/A	>0.05 (ANOVA/Tukey test)	349	1705.8	N/A
Numbers of patients with significant change in % DLCO	N/A	53(50)	N/A	N/A	N/A	N/A	N/A	81(58)	N/A	N/A	N/A	N/A	0.3083 (*χ* ^2^-test with Yates correction)	1	1.038	0.6328 to 1.126

Continuous normally distributed variables are depicted as mean ± standard deviation (SD) or continuous non-normally distributed variables are depicted as median with Q3–Q1 in parenthesis or categorial variables are depicted as frequencies with percentages in parenthesis.

FVC: Forced vital capacity, DLCO: Diffusing capacity of the lungs for carbon monoxide, BL: before intervention(s), EL: after complete of treatment.

All results are considered significant if the *p*-value was less than 0.05.

A significant change in pulmonary function tests was defined as >10% change in % FVC or >15% change in % DLCO, in conformity with the ATS/ERS guideline.

N/A: Not applicable, df: Degree of freedom, CI: confidence interval (using the approximation of Katz. For categorial variable).

Test value (*F*-value for ANOVA, Kruskal-Wallis Statistic for Kruskal-Wallis’ test; *χ* ^2^-value for *χ* ^2^-test; relative risk for Fisher’s exact test).

### 3.4 Adverse effects

Nausea, vomiting, and appetite loss were common treatment-related adverse effects observed in the CYC and MMF cohorts. Cyclophosphamide-induced leukopenia and thrombocytopenia are treatment-related side effects. The details of treatment-related non-serious side effects within the treatment(s) and follow-up periods are reported in Table 3.

**Table 3:** Treatment-related non-serious side effects within treatment(s) and follow-up period

Effect	Cohorts				
	MMF	CYC			
Treatment	Mycophenolate mofetil + prednisolone	Cyclophosphamide + prednisolone	Comparisons between cohorts		
Patients	105	140	*p*-value	Relative risk	95% CI
Leukopenia (white blood cells <2,500 × 10^3^/mL)	8(8)	55(39)^*^	<0.0001	0.2383	0.1229 to 0.4618
Neutropenia (neutrophils <1,000 × 10^3^/mL)	5(5)	11(8)	0.4363	0.4363	0.3409 to 1.502
Anemia (Hemoglobin <10 gm/dl)	12(11)	29(21)	0.0588	0.642	0.3898 to 1.057
Thrombocytopenia (Platelets <10^6^/mL)	2(2)	11(8)^*^	0.0458	0.3465	0.09603 to 1.250
Hematuria	4(4)	12(9)	0.1917	0.5668	0.2395 to 1.341
Pneumonia	9(9)	15(11)	0.6669	0.8633	0.5040 to 1.479

Variables are depicted as frequencies with percentages in parenthesis.

^*^Cyclophosphamide-emergent adverse effects.

Fisher exact test was used for statistical analysis.

All results are considered significant if the *p*-value was less than 0.05.

CI: confidence interval (using the approximation of Katz).

There were more serious adverse effects and deaths related to cyclophosphamide treatment(s) than mycophenolate mofetil during the treatment(s) and follow-up periods. In addition, only four (4%) patients from the MMF cohort and six (4%) patients from the CYC cohort had pulmonary hypertension within the treatment(s) and follow-up period. The details of serious adverse effects due to treatment(s) and disease(s) within the treatment(s) and follow-up periods are reported in Table 4.

**Table 4:** Serious adverse effects due to treatment(s) and disease(s) within treatment(s) and follow-up period

Effect	Cohorts				
	MMF	CYC			
Treatment	Mycophenolate mofetil + prednisolone	Cyclophosphamide + prednisolone	Comparisons between cohorts		
Patients	105	140	*p*-value	Relative risk	95% CI
Serious adverse effects related to treatment(s)	6(6)	20(14)^*^	0.0362	0.5105	0.2492 to 1.046
Serious adverse effects related to underlying disease(s)	17(16)	29(21)	0.4113	0.8357	0.5554 to 1.257
Pulmonary hypertension	4(4)	6(4)	0.9999	0.9307	0.4294 to 2.017
Death due to any cause(s)	1(1)	9(6)^*^	0.0468	0.226	0.03499 to 1.459

Variables are depicted as frequencies with percentages in parenthesis.

Fisher exact test was used for statistical analyses.

Pulmonary artery systolic pressure >50 mm Hg measured by cardiac color ultrasound, or mean pulmonary artery pressure >20 mm Hg measured by right heart catheterization.

All results are considered significant if the *p*-value was less than 0.05.

Hospitalization or life-threatening effects were considered as serious adverse effects related to treatment(s).

Hospitalization or life-threatening effects due to persistent disease was considered as serious adverse effects related to underlying disease(s).

^*^Cyclophosphamide-emergent adverse effects.

CI: confidence interval (using the approximation of Katz).

## 4 Discussion

% FVC and the number of patients with ≥70% FVC were increased for patients of the CYC cohort than their BL condition and those patients of the MMF at EL. FVC increase in MMF and CYC cohorts was approximately 8.57 and 15.07%, respectively. In addition, numbers of patients with significant change in % FVC were more in the CYC cohort than those of the MMF cohort. The % FVC results in the current study were consistent with those of a Cochrane database of systematic reviews [2] but were not consistent with those of a trial on patients with scleroderma-related ILD [5]. The patients in the trial [5] also received treatment for scleroderma, for example, intervention(s) with rituximab, tocilizumab, immunoglobulin, and/or azathioprine. In addition, baseline demographic and clinical conditions affect the treatment(s) of CTD-ILD [5]. Cyclophosphamide treatment(s) has a superior effect in the improvement of % FVC than those of mycophenolate mofetil in patients with CTD-ILD.

There is no statistical difference between the cohorts for changes in DLCO value. The % DLCO results in the current study were not consistent with those of a Cochrane database of systematic reviews [2] and a trial on patients with scleroderma-related ILD [5]. Mycophenolate mofetil 2 years treatment(s) and cyclophosphamide treatment(s) both have beneficial effects on gas transfer in patients with CTD-ILD.

Patients in the CYC cohort had higher rates of leukopenia, thrombocytopenia, and serious adverse effects related to treatment(s) than those in the MMF cohort. The results of the adverse effects of the current study are consistent with those of a trial on patients with scleroderma-related ILD [5] and a Cochrane database of systematic reviews [2]. Mycophenolate mofetil for 2 years treatment(s) is less toxic and better tolerated than cyclophosphamide treatment(s) in patients with CTD-ILD. The primary disease and/or the comorbidities (e.g., history of seizures requiring anti-epileptic drugs) which have a confounding effects on severe adverse events and treatment related death.

Patients in the CYC cohort had higher mortality rates than those in the MMF cohort did. However, only 10 (4%; 4% each from both cohorts) had pulmonary hypertension. There are many factors that affect pulmonary arterial pressure, such as systemic sclerosis is prone to pulmonary arterial hypertension, and other rheumatic diseases are less. The results of pulmonary hypertension and death in the follow-up of the current study are consistent with those of a trial on patients with scleroderma-related ILD [5]. Mycophenolate mofetil has positive effects on pulmonary hypertension [16]. Positive effects on pulmonary hypertension, especially in patients with <70% FVC, have survival advantages [17]. In addition, the mortality was higher in the CYC cohort due to adverse effects of cyclophosphamide. Mycophenolate mofetil may have positive effects on pulmonary hypertension and ultimately on the survival of patients with CTD-ILD.

Unlike other trials [5,17], the current study used ‘number of patients with an ≥70% FVC’ and ‘number of patients with an ≥75% DLCO’ as an outcome measurement. Because the number of patients with an FVC above 70% depends on the FVC at baseline BT, the same goes for ≥75% DLCO. For example, if a patient starts with an FVC of 50%, they will need an increase of 20% while a patient who starts with an FVC of 69% will need only 1% increase of FVC. So therefore, the increase of FVC and the increase of DLCO in both cohorts are the only good outcome parameter to describe efficacy of treatment and the number of patients with an FVC of ≥70 and ≥75% DLCO is not. Furthermore, the absolute FVC and DLCO at BL condition in both cohorts are also dependent on the baseline numbers and has nothing to do with the effect of treatment. FVC ≥70% [18] and DLCO ≥75% [19] are normal values for these pulmonary physiological parameters. These parameters are affected by disease conditions [5]. In addition, most of patients had FVC about 60% and DLCO about 63% at BL. Thus, by evaluating these parameters (‘number of patients with an ≥70% FVC’ and ‘number of patients with an ≥75% DLCO’), treatment(s) efficacy can be evaluated.

Unlike in a trial on patients with scleroderma-related ILD [5], the number of deaths due to cyclophosphamide treatment was lower in the current study (6 vs 17%). Clinicians have realized the side effects of cyclophosphamide in the past 2 years. One is that the medication dosage control is better than before, the patient’s reexamination and monitoring are also in place, and some adverse reactions can be observed over time. Therefore, the mortality rate was not as high as before (50 mg/day oral dose).

Oral cyclophosphamide 50 mg once every other day (the cumulative dose of cyclophosphamide should not exceed 10 g) used instead of the once-daily 50 mg oral dose. This is institutional protocol to reduce toxic effects of cyclophosphamide.

Patients who stopped therapy excluded from analysis. This is causing a selection bias. Patients who discontinued cyclophosphamide or mycophenolate mofetil were not useful to evaluate the efficacy parameters of cyclophosphamide or mycophenolate mofetil. Therefore, patients excluded from analysis.

It is valuable to compare the efficacy and safety of two potent immunosuppressive drugs in real life; however, there are some issues that can influence drawing conclusions from the study, e.g., the study is retrospective and the lack of randomized trials. This study compared the one-year treatment(s) of cyclophosphamide with 2-year treatment(s) of mycophenolate mofetil. However, this is technically unusual. The study included Han Chinese patients only, which makes issue of generalizability of the results. The possible justification for the same is that there are basically no other people race (Mongolian and Tibetan) residents of people in Taiyuan, Shanxi. All residents are Han Chinese.

## 5 Conclusions

Cyclophosphamide plus prednisolone treatment(s) has a superior effect in the improvement of % FVC than those of treatment(s) of mycophenolate mofetil plus prednisolone in patients with CTD-ILD. Mycophenolate mofetil plus prednisolone treatment(s) and cyclophosphamide plus prednisolone treatment(s) have beneficial effects on gas transfer in patients with CTD-ILD. Mycophenolate mofetil plus prednisolone for 2-year treatment(s) is less toxic and better tolerated and has positive effects on the survival of patients than cyclophosphamide plus prednisolone in patients with CTD-ILD. By evaluating the number of patients with ≥70% FVC and ≥75% DLCO treatment(s), efficacy could be evaluated. Oral cyclophosphamide 50 mg once every other day (the cumulative dose of cyclophosphamide should not exceed 10 g) has fewer death than previously reported studies on cyclophosphamide (once daily 50 mg oral cyclophosphamide for 1 year).

## Abbreviations

CTD: Connective tissue disease
ILD: Interstitial lung disease
CTD-ILD: Connective tissue disease-associated interstitial lung disease
*χ* ^2^ test: Chi-square test
FVC: Forced vital capacity
DLCO: Diffusing capacity of the lungs for carbon monoxide
ANOVA: Analysis of variance
SD: Standard deviation
BL: Before intervention(s)
EL: after complete of treatment
MMF cohort: Patients received oral 0.5 g twice a day of mycophenolate mofetil for 2 years + oral prednisolone
CYC cohort: Patients received oral cyclophosphamide 50 mg once every other day; the cumulative dose of cyclophosphamide should not exceed 10 g + oral prednisolone
ATS: The American Thoracic Society
ERS: European Respiratory Society.

## References

[1] Antoniou KM, Margaritopoulos GA, Tomassetti S, Bonella F, Costabel U, Poletti V. Interstitial lung disease. Eur Respir Rev. 2014;23(131):40–54. 10.1183/09059180.00009113. PMC9487254 24591661

[2] Barnes H, Holland AE, Westall GP, Goh NS, Glaspole IN. Cyclophosphamide for connective tissue disease-associated interstitial lung disease. Cochrane Database Syst Rev. 2018;1(1):CD010908. 10.1002/14651858.CD010908.pub2. PMC6491200 29297205

[3] Ahlmann M, Hempel G. The effect of cyclophosphamide on the immune system: implications for clinical cancer therapy. Cancer Chemother Pharmacol. 2016;78(4):661–71. 10.1007/s00280-016-3152-1. 27646791

[4] Furst DE, Tseng CH, Clements PJ, Strange C, Tashkin DP, Roth MD, et al. Adverse events during the scleroderma lung study. Am J Med. 2011;4(5):459–67. 10.1016/j.amjmed.2010.12.009. 21531236

[5] Tashkin DP, Roth MD, Clements PJ, Furst DE, Khanna D, Kleerup EC, et al. Mycophenolate mofetil versus oral cyclophosphamide in scleroderma-related interstitial lung disease (SLS II): A randomised controlled, double-blind, parallel group trial. Lancet Respir Med. 2016;4(9):708–19. 10.1016/S2213-2600(16)30152-7. PMC5014629 27469583

[6] Staatz CE, Tett SE. Pharmacology and toxicology of mycophenolate in organ transplant recipients: an update. Arch Toxicol. 2014;88(7):1351–89. 10.1007/s00204-014-1247-1. 24792322

[7] Hahn BH, McMahon MA, Wilkinson A, Wallace WD, Daikh DI, Fitzgerald JD, et al. American College of Rheumatology guidelines for the screening, treatment, and management of lupus nephritis. Arthritis Care Res (Hoboken). 2012;64(6):797–808. 10.1002/acr.21664. PMC3437757 22556106

[8] Simeón-Aznar CP, Fonollosa-Plá V, Tolosa-Vilella C, Selva-O’Callaghan A, Solans-Laqué R, Vilardell-Tarrés M. Effect of mycophenolate sodium in scleroderma-related interstitial lung disease. Clin Rheumatol. 2011;30(11):1393–8. 10.1007/s10067-011-1823-1. 21881859

[9] Fischer A, Brown KK, Du Bois RM, Frankel SK, Cosgrove GP, Fernandez-Perez ER, et al. Mycophenolate mofetil improves lung function in connective tissue disease-associated interstitial lung disease. J Rheumatol. 2013;40(5):640–6. 10.3899/jrheum.121043. PMC3676865 23457378

[10] Ueda T, Sakagami T, Kikuchi T, Takada T. Mycophenolate mofetil as a therapeutic agent for interstitial lung diseases in systemic sclerosis. Int Respir Investig. 2018;56(1):14–20. 10.1016/j.resinv.2017.11.004. 29325675

[11] Dellaripa PF. Interstitial lung disease in the connective tissue diseases: A paradigm shift in diagnosis and treatment. Clin Immunol. 2018;186:71–3. 10.1016/j.clim.2017.09.015. 28923440

[12] Khanna D, Lin CJF, Furst DE, Wagner B, Zucchetto M, Raghu G, et al. Long-term safety and efficacy of tocilizumab in early systemic sclerosis-interstitial lung disease: Open-label extension of a phase 3 randomized controlled trial. Am J Respir Crit Care Med. 2022;205(6):674–84. 10.1164/rccm.202103-0714OC. 34851799

[13] Vij R, Strek ME. Diagnosis and treatment of connective tissue disease-associated interstitial lung disease. Chest. 2013;143(3):814–24. 10.1378/chest.12-0741. PMC3590889 23460159

[14] Quan XY, Chen HT, Liang SQ, Yang C, Yao CW, Xu YZ, et al. Revisited cyclophosphamide in the treatment of lupus nephritis. Biomed Res Int. 2022;2022:8345737. 10.1155/2022/8345737. PMC9192236 35707391

[15] Peredo RA, Mehta V, Beegle S. Interstitial lung disease associated with connective tissue diseases. Adv Exp Med Biol. 2021;1304:73–94. 10.1007/978-3-030-68748-9_5. 34019264

[16] Zheng Y, Li M, Zhang Y, Shi X, Li L, Jin M. Effects and mechanisms of mycophenolate mofetil on pulmonary arterial hypertension in rats. Rheumatol Int. 2010;30(3):341–8. 10.1007/s00296-009-0966-8. 19466418

[17] Saketkoo LA, Lammi MR, Fischer A, Molitor J, Steen VD. on behalf of the PHAROS Investigators. SAT0442 Mycophenolate Mofetil (MMF) use in scleroderma patients with pulmonary hypertension: FVC, outcomes and survival- observations from the Pulmonary Hypertension Recognition and Outcomes in Scleroderma (Pharos) cohort. Ann Rheum Dis. 2015;74:820. 10.1136/annrheumdis-2015-eular.3483.

[18] Jeong WG, Kim YH, Lee JE, Oh IJ, Song SY, Chae KJ, et al. Predictive value of interstitial lung abnormalities for postoperative pulmonary complications in elderly patients with early stage lung cancer. Cancer Res Treat. 2022;54(3):744–52. 10.4143/crt.2021.772. PMC9296932 34583454

[19] Nguyen LP, Harper RW, Louie S. Using and interpreting carbon monoxide diffusing capacity (DLCO) correctly. Consultant. 2016;6(5):440–5, https://www.consultant360.com/articles/using-and-interpreting-carbon-monoxide-diffusing-capacity-dlco-correctly.

